# Replication Factor C Complexes Play Unique Pro- and Anti-Establishment Roles in Sister Chromatid Cohesion

**DOI:** 10.1371/journal.pone.0015381

**Published:** 2010-10-27

**Authors:** Marie E. Maradeo, Robert V. Skibbens

**Affiliations:** Department of Biological Sciences, Lehigh University, Bethlehem, Pennsylvania, United States of America; National Cancer Institute, United States of America

## Abstract

Recent studies have lead to a rapid expansion of sister chromatid cohesion pathways. Of particular interest is the growth in classifications of anti-establishment factors—now including those that are cohesin-associated (Rad61/WAPL and Pds5) or DNA replication fork-associated (Elg1-RFC). In this study, we show that the two classes of anti-establishment complexes are indistinguishable when challenged both genetically and functionally. These findings suggest that both classes function in a singular pathway that is centered on Ctf7/Eco1 (herein termed Ctf7) regulation. The anti-establishment activity of Elg1-RFC complex is particular intriguing given that an alternate Ctf18-RFC complex exhibits robust pro-establishment activity. Here, we provide several lines of evidence, including the use of Ctf7 bypass suppressors, indicating that these activities are not simply antagonistic. Moreover, the results suggest that Ctf18-RFC is capable of promoting sister chromatid pairing reactions independent of Ctf7. The combination of these studies suggest a new model of sister chromatid pairing regulation.

## Introduction

The goal of cell division is to faithfully replicate the genome and then segregate the resulting sister chromatids into the newly forming daughter cells. The time between chromosome replication and sister chromatid segregation can be quite significant. Thus, a major challenge for the cell is to identify over time the products of chromosome replication as sister chromatids. This feat is accomplished by tethering together each sister pair – a multi-step process collectively termed cohesion [1]. In budding yeast, sister chromatid associations are maintained by cohesin complexes that contain Mcd1/Scc1, Smc1, Smc3 and Irr1/Scc3 [2]–[4]. In vertebrate cells, Sororin is also required for cohesion maintenance [5], [6], revealing that cohesin structure is likely quite complex [7]. The deposition of cohesins onto chromosomes occurs through a separate complex composed of Scc2 and Scc4 [8]. Notably, cohesin and its deposition onto DNA are not sufficient to tether together sister chromatids. Instead, chromatin-associated cohesins must be converted to a paired stated by the cohesion establishment factor Ctf7 [9], [10]. Ctf7 is an acetyltransferase that modifies Smc3 specifically during S-phase – a modification that may be coupled to passage of the DNA replication fork [9], [11]–[14]. In response to DNA damage, however, Ctf7 becomes active during G_2_/M. In this instance, Ctf7 acetylates Mcd1/Scc1 to promote sister chromatid pairing and can do so independent of DNA repair/replication factors [15]–[19].

How do cells limit DNA pairing reactions to sister chromatids? An early but still popular model posits that Ctf7 interacts with or even rides the replication fork to coordinate the emergence of nascent sister chromatids from the DNA replisome to conversion of cohesins to a paired state [20]. This model is based on genetic interactions between *CTF7* and *CTF18* and *POL30* [9]. Ctf18 associates with other Replication Factor C (RFC) subunits (Rfc2-Rfc5 and Dcc1 and Ctf8) to load Proliferating Cell Nuclear Antigen (PCNA) sliding clamps onto primed DNA [21], [22]. Subsequent Ctf7 binding studies and identification of numerous DNA replication factors that promote efficient cohesion (including PCNA, RFC complexes, Chl1, Tof1, Csm3 and Rad27/Fen1) support the view that cohesion is coupled to DNA replication [9], [23]–[33]. Not only are DNA replication factors crucial for sister chromatid pairing, but mutations in cohesion factors can produce transient DNA replication fork pauses [34]. Thus, cohesion establishment and DNA replication fork progression appear intimately entwined.

Anti-establishment factors are in part defined by the observation that their deletion (or diminished function) rescues conditional growth and cohesion defects associated with *ctf7* mutations [19]. Currently, anti-establishment factors fall into two categories: those that are cohesin-associated (Pds5 and Rad61) and those that are DNA replication fork-associated (Elg1-RFC) [19], [27], [28], [35]–[37]. Consistent with their proposed sites of actions, the mechanisms through which these anti-establishment factors function are thought to be quite different. As cohesin-associated factors, Pds5 and Rad61 are posited to act directly on cohesins - promoting cohesin-chromatin dynamics up until Ctf7-dependent acetylation of Smc3. In contrast, fork-associated factors such as Elg1-RFC are thought to regulate Ctf7 function – possibly through sequestration or inactivation. Given numerous studies that now directly link defects in cohesion pathways to aneuploidy and cancer (breast cancer and aggressive melanoma) and developmental defects (including Cornelia de Lange Syndrome, Roberts Syndrome/SC-phocomelia and Warsaw Breakage Syndrome), characterization of this newest class of anti-establishment factors becomes of great interest [38], [39]. Here, we report new evidence that is relevant to mechanisms through which establishment and anti-establishment factors regulate cohesion.

## Results

### Ctf18-RFC performs cohesion functions separate from Ctf7-dependent acetylation of Smc3

Ctf18 physically associates with Ctf7 *in vitro* and both *ctf18* yeast mutant cells and human cells reduced in Ctf18 levels exhibit cohesion defects [23], [30], [31], [34]. In yeast, *CTF18* deletion exacerbates *ctf7* mutant cell growth defects to the point of lethality [9], all of which position Ctf18-RFC as a pro-establishment complex [19]. In turn, the only essential function of Ctf7 is to acetylate Smc3 during S-phase such that the *smc3* acetylmimetic allele *smc3^K113Q^* (herein termed *smc3Q*) suppresses *ctf7* mutant strain phenotypes [12]–[14]. We decided to exploit this synthetic lethality and *smc3^Q^*-dependent bypass of Ctf7 function to test whether Ctf18-RFC functions directly through Ctf7 activation. Cells expressing *smc3^Q^* no longer contain the essential lysine target of Ctf7. If Ctf18-RFC functions directly through Ctf7, then *smc3^Q^* should not only bypass *ctf7* mutant cell phenotypes but also rescue *ctf7 ctf18* synthetic lethality. To test this notion, *ctf7-203 smc3^Q^* cells were crossed to *ctf18* deletion cells and the resulting diploids sporulated. We recovered the appropriate number of *ctf7*, *ctf18* and *smc3^Q^* single mutant spores and also *ctf7 smc3^Q^* and *ctf18 smc3^Q^* double mutant spores (Table 1). As expected, no viable *ctf7 ctf18* double mutant spores were recovered, confirming previous results [9]. Despite the ability of *smc3^Q^* to bypass the requirement for Ctf7-dependent Smc3 acetylation under these conditions, triple mutant *ctf7 ctf18 smc3^Q^* cells were never recovered even after multiple attempts and from independent crosses. The inability to recover triple mutant *ctf7 ctf18 smc3^Q^* cells is not due to adverse genetic interactions between *ctf18* and *smc3^Q^* since these double mutants were recovered at the expected frequency (Table 1). Results that *smc3^Q^* fails to bypass *ctf7 ctf18* lethality suggest that Ctf18-RFC promotes cohesion in a fashion separate from Ctf7-dependent acetylation of Smc3.

**Table 1:** *smc3* acetyl mimics can not bypass *ctf7-203 ctf18* synthetic lethality.

	Observed	Expected
Wild Type	8	13.5
*ctf7-203*	18	13.5
*ctf18*	16	13.5
*smc3^K113Q^*	19	13.5
*ctf7-203 smc3^K113Q^*	11	13.5
*ctf18 smc3^K113Q^*	10	13.5
*ctf7-203 ctf18*	0	13.5
*ctf18 ctf7-203 smc3^K113Q^*	0	13.5
Dead	26	0

Cells harboring *ctf7-203* mutation along with *smc3^K113Q^* acetyl mimic were crossed to *ctf18* knockout cells. Diploids were sporulated, dissected and tetrads analyzed. Genotypes obtained from this cross are located in the observed column. Results reflect analysis from strain YMM506 crossed to strain YMM705.

**Figure 1:**
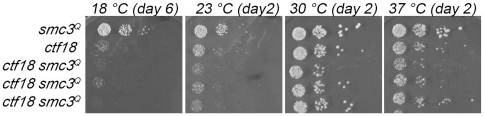
*SMC3* acetylation mimetic provides no growth benefit to *ctf18* mutant cells. 10 fold serial dilutions of *smc3^Q^* and *ctf18* single mutant cells and three independent isolates of *ctf18 smc3^Q^* double mutant cells. Colony growth shown for cells on rich medium plates grown at 18°C, 23°C, 30°C, and 37°C for number of days indicated. Strains shown include YMM872, YMM873, YMM874, YMM875 and YMM 876.

The notion that Ctf18-RFC promotes cohesion independent of Ctf7 is novel. To further test this model, we pursued three additional independent lines of inquiry. In the first, we reasoned that if Ctf18-RFC functions independent of Smc3 acetylation, then the *smc3^Q^* should fail to suppress *ctf18* mutant cell growth defects. On the other hand, a finding that *smc3^Q^* rescues *ctf18* mutant cell growth defects would indicate that Ctf18 functions through Ctf7-dependent acetylation of Smc3. Serial dilution of log phase *smc3^Q^* and *ctf18* single mutant cells and *smc3^Q^ ctf18* double mutant cells were plated onto rich medium and challenged at a range of temperatures. The results reveal that the addition of *smc3^Q^* did not rescue the slow growth phenotype of *ctf18* strains (Figure 1), separating out the pro-establishment function of Ctf18-RFC from that of Ctf7. Nor did *smc3^Q^* exacerbate *ctf18* null cell growth, obviating concerns that the combination of *ctf18* and *smc3^Q^* adversely affects other cellular pathways such as DNA replication fork stability/progression [34].

Second, we took advantage of prior studies that deletion of *ELG1* suppresses both *ctf7* mutant cell conditional growth and sister chromatid defects, identifying Elg1-RFC as an anti-establishment factor that likely directly opposes Ctf7 function [27], [28]. If Ctf18-RFC is not simply antagonistic to Elg1-RFC, then combining *elg1* and *ctf18* deletions in *ctf7 smc3^Q^* mutant cells should produce inviable cells: ie., while *smc3^Q^* bypasses *ctf7* cohesion defect, deletion of anti-establishment *ELG1* will fail to compensate for deletion of pro-establishment *CTF18*. Analysis of *ctf7 smc3^Q^ elg1* crossed to *ctf18 smc3^Q^* produces exactly this result. Despite numerous attempts, we were unable to obtain viable *ctf7 ctf18 elg1 smc3^Q^* mutant cell lines (Table 2).

**Table 2:** *smc3* acetyl mimics and elg1 deletion can not bypass *ctf7-203 ctf18* synthetic lethality.

	Observed	Expected
*smc3^K113Q^*	24	23
*smc3^K113Q^ ctf7-203*	14	23
*smc3^K113Q^ ctf18*	14	23
*smc3^K113Q^ elg1*	23	23
*smc3^K113Q^ ctf7-203 elg1*	21	23
*smc3^K113Q^ ctf7-203 ctf18*	0	23
*smc3^K113Q^ elg1 ctf18*	16	23
*smc3^K113Q^ ctf7-203 elg1 ctf18*	0	23
Dead	72	0

Cells harboring *ctf7-203* mutation along with *smc3^K113Q^* acetyl mimic and *elg1* deletion were crossed to *ctf18* knockout cells carrying *smc3^K113Q^* acetyl mimic. Diploids were sporulated, dissected and tetrads analyzed. Genotypes obtained from this cross are located in the observed column. Results reflect analysis from strain YMM784 crossed to strain YMM737.

**Figure 2:**
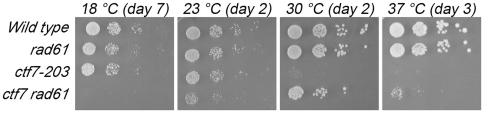
*rad61* deletion provides limited bypass of *ctf7* mutant cell lethality. 10 fold serial dilutions of wild type, *rad61* and *ctf7-203* single mutant cells and also *ctf7 rad61* double mutant cells. Colony growth shown for cells on rich medium plates grown at 18°C, 23°C, 30°C, and 37°C for number of days indicated. Strains shown include YBS255, YMM808, YBS514 and YMM828.

The third test of the model that Ctf18 promotes cohesion independent of Ctf7 is predicated on *RAD61* (WAPL in higher eukaryotes). Ctf7 is essential for cell viability, though recent findings reveal that *ctf7* null cells are viable if also deleted for *RAD61* [35], [36]. If correct, our model that Ctf18 can promote sister chromatid pairing independent of Ctf7 predicts that *rad61 ctf7* cells will become inviable upon the additional deletion of *CTF18*. We first confirmed *rad61* deletion bypass of Ctf7 function. Consistent with prior reports, sporulation of *ra61/RAD61*and *ctf7/CTF7* heterozygous diploids produced viable *rad61 ctf7* double mutant cells. We then compared growth of wildtype, single and double mutant strains under a range of temperatures (18°C, 23°C, 30°C and 37°C). *ctf7-203* was included in this analysis given that *CTF7* deletion renders cells inviable [9]. As expected, wildtype and *rad61* cells exhibit robust growth at all temperatures while *ctf7-203* mutant cells are inviable at 30°C and above (Figure 2). In contrast, *rad61 ctf7* double mutant cells exhibit robust growth at 30°C, although these cells are growth inhibited at 18° and exhibit at least modest growth defects at both 23°C and 37°C (Figure 2). Thus, this analysis uncovered unanticipated limitations regarding *rad61* bypass of Ctf7 function but confirm *rad61*-bypass of *ctf7* null cell lethality at a wide range of temperatures.

**Figure 3:**
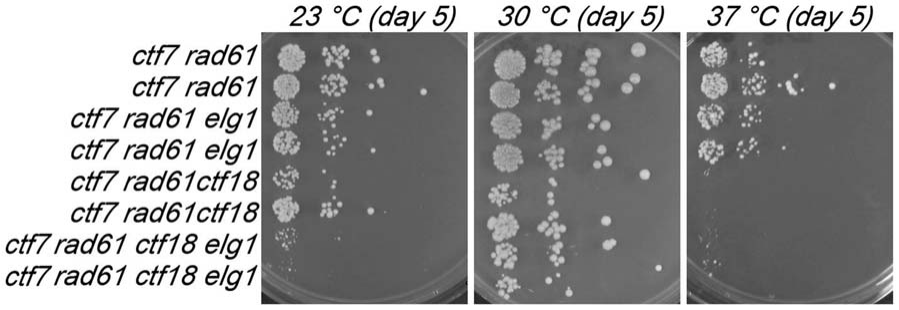
Neither *rad61* nor *elg1* deletion, nor the combination, rescue *ctf18* deficiency in *ctf7* mutant cells. 10 fold serial dilutions of *ctf7 rad61* double, *ctf7 rad61 elg1* and *ctf7 rad61 ctf18* triple mutant cells and also *ctf7 rad61 ctf18 elg1* quadruple mutant cells. Colony growth shown for cells on rich medium plates grown at 23°C, 30°C and 37°C for the number of days indicated. Strains shown include YMM828, YMM829, YMM821, YMM823, YMM820, YMM827, YMM822 and YMM824.

Having identified the range of conditions that support *rad61*-bypass of *ctf7* null cell lethality, we tested the prediction that *rad61* would fail to rescue *ctf7 ctf18* mutant cell growth defects. *ctf18* single mutant cells were crossed to *ctf7 rad61* double mutant cells and the resulting diploids sporulated. We also included *elg1* mutation within these crosses (see below). *ctf7 rad61 ctf18* triple mutant cells were recovered from these crosses. However, these triple mutant cells are inviable at temperatures that otherwise support *ctf7 rad61* mutant cell growth (Figure 3). Given that *rad61* bypasses completely *ctf7* null cell lethality under these conditions, the finding that *rad61* deletion is not sufficient to suppress *ctf7 ctf18* mutant cell conditional lethality is consistent with the model that Ctf18-RFC exhibits establishment activities beyond those associated with Ctf7.

We also recovered *ctf7 rad61 elg1* triple mutant cells and *ctf7 rad61 ctf18 elg1* quadruple mutant cells. The results show that the adverse effect of *ctf18* deletion from *ctf7 rad61* cells is not a general property of diminished RFC function: *ctf7 rad61 elg1* triple mutant cell growth was identical to that exhibited by *ctf7 rad61* double mutant cells (Figure 3). Intriguingly, *elg1* deletion failed to provide any benefit to *ctf7* mutant cells beyond those already conferred by *rad61*: *ctf7 rad61* cells grew similar to *ctf7 rad61 elg1* cells and *ctf7 rad61 ctf18* cells grew similar to *ctf7 rad61 ctf18 elg1* cells (Figure 3).

### *ctf7 rad61 ctf18* mutant cells progress through S-phase similar to wildtype cells

**Figure 4:**
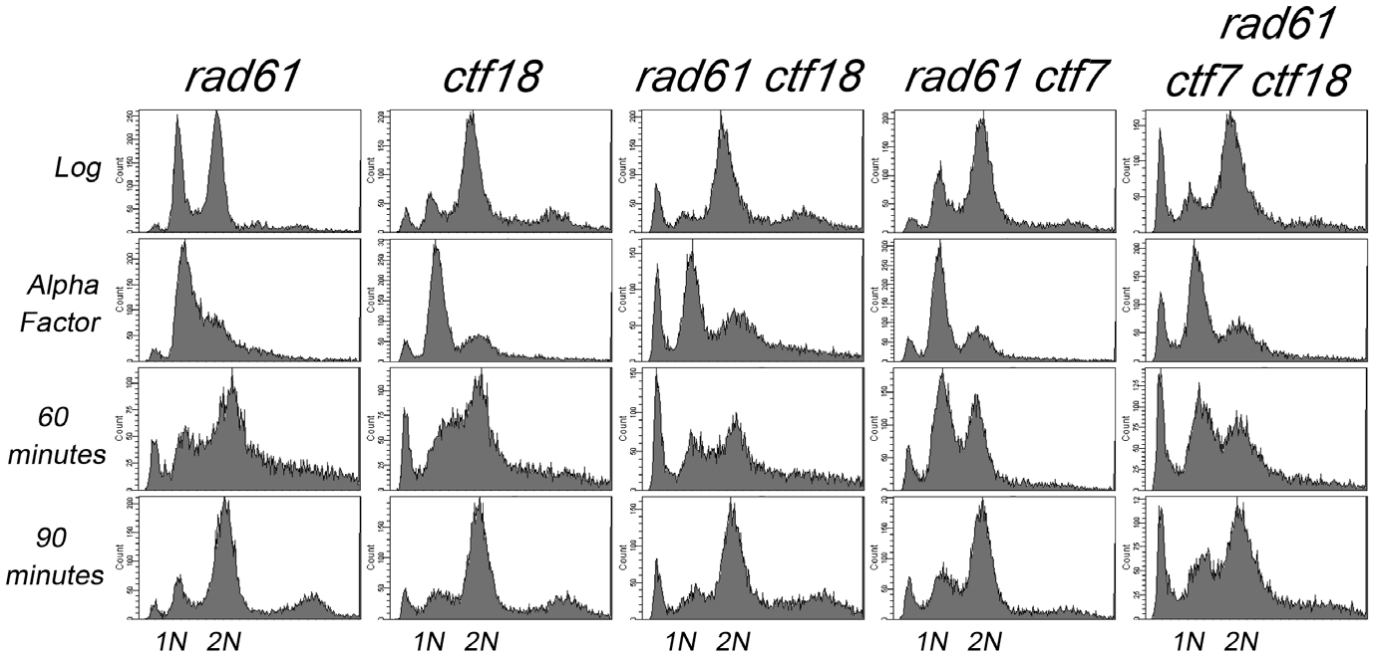
*rad61 ctf7 ctf18* triple mutant cells progress normally through S phase. DNA profiles of *rad61* and *ctf18* single mutant cells and *rad61 ctf18* and *rad61 ctf7* and also *rad61 ctf7 ctf18* triple mutant strains during log phase growth (Log), synchronized in G_1_ (α-Factor) at 30°C and then released into fresh medium at 37°C. Time points after release into fresh medium indicated. Strains shown include YMM808, YBS1160, YMM813, YMM829 and YMM825.

The roles of Ctf7, Rad61 and Ctf18-RFC in cohesion are well documented. Here, we address whether the conditional nature of *ctf7 rad61 ctf18* triple mutant cells described above is instead due to DNA replication defects. We released G_1_-arrested and synchronized *ctf7 rad61 ctf18* triple mutant cultures into rich medium shifted to the restrictive temperature of 37°C. We included in our analyses *rad61* and *ctf18* single mutant cells and also *rad61 ctf18* double mutant cells. Even during log phase growth, *ctf7 rad61 ctf18* triple mutant cells exhibited a mitotic delay similar to that of *ctf7* mutant cells. Prior findings revealed that the G_2_/M delay in *ctf7* cells requires the mitotic spindle checkpoint but not DNA damage or unreplicated DNA checkpoints [9]. Upon release from G_1_, wildtype, *rad61* and *ctf18* single mutant cells and *rad61 ctf18* double mutant cells and also *ctf7 rad61 ctf18* triple mutant cells all exited S-phase in synchrony such that the time interval from G_1_ to mid-replication and then to G_2_/M accumulation is nearly identical (Figure 4). These kinetics are in stark contrast to S-phase progression in DNA replication mutants – which often require over 4X the time interval between G_1_ to G_2_/M [40]. While these studies can not rule out transient fork progression defects, at present we find no evidence that *ctf7 rad61 ctf18* triple mutants exhibit gross S-phase progression defects, consistent with the notion that the conditional lethality of these cells is likely a result of increased cohesion defects.

### Elg1-RFC and Rad61 operate through a common anti-establishment mechanism that opposes Ctf7 function

**Figure 5:**
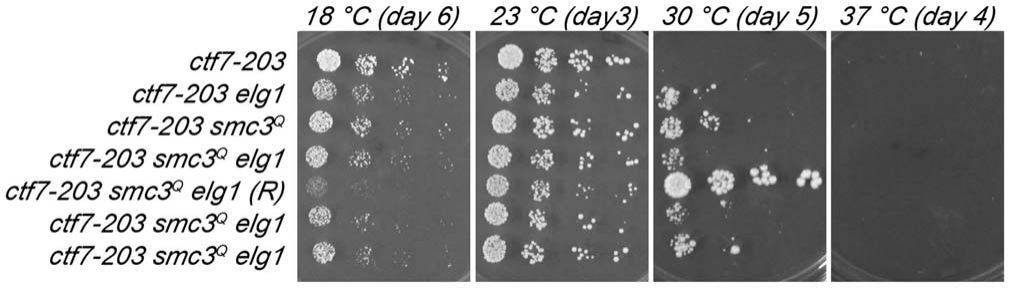
*elg1* deletion and *smc3* acetylmimetic alleles exhibit similar effects on *ctf7* mutant cells. 10 fold serial dilutions of *ctf7-203* single mutant cells, ctf7-203 elg1 and *ctf7-203 smc3^Q^* double mutant cells and four independent isolates of *ctf7-203 smc3^Q^ elg1* triple mutant cells. Colony growth shown for cells on rich medium plates grown at 18°C, 23°C, 30°C and 37°C for the number of days indicated. Revertant (R) triple mutant cell shown. Strains shown include YMM865, YMM866, YMM867, YMM869, YMM870 and YMM871.

The above finding that *elg1* deletion fails to provide any benefit to *ctf7* mutant cells beyond those already conferred by *rad61* suggests that Rad61 and Elg1-RFC regulate cohesion through a singular mechanism – specifically in opposition to Ctf7 function. To further test this hypothesis, we again turned to *smc3^Q^* suppression of *ctf7* mutant cells to assess the role of *elg1* in suppressing *ctf7* mutant cell phenotypes [12]–[14]. *ctf7-203 smc3^Q^* and *elg1* cells were mated and the resulting diploids sporulated to generate the desired single, double and triple mutant strains. Log phase growth of *ctf7* single mutant strains was compared to that of double (*ctf7 smc3^Q^* and *ctf7 elg1*) mutant strains by serial dilution and at a range of temperatures (18°C, 23°C, 30°C and 37°C). As expected, *ctf7* mutant cells were inviable at temperatures tested above 23°C whereas *ctf7* mutant cells coupled with either *elg1* or *smc3^Q^* remained viable up to 30°C (Figure 5). The *smc3* acetylmimetic bypass allele did not outperform *elg1* deletion in suppressing *ctf7* mutant cell growth defects at any temperature assayed, but neither provided for complete bypass of Ctf7 function (Figure 5).

Second, we tested the prediction that if Elg1-RFC opposes directly Ctf7-dependent Smc3 actylation, then deletion of *ELG1* should fail to provide an additional growth benefit to *ctf7* mutant cells also expressing *smc3^Q^*. On the other hand, if Elg1-RFC opposes sister chromatid pairing reactions downstream of Ctf7, then *ELG1* deletion should provide added growth benefit when placed in *ctf7 smc3^Q^* mutant cells. To differentiate between these two modes of Elg1-RFC anti-establishment activity, we obtained triple mutant *ctf7 smc3^Q^ elg1* strains from the crosses described above and at the expected frequency. Results from serial dilutions show that the additional deletion of *ELG1* failed to provide any growth benefit to *ctf7 smc3^Q^* double mutant cells across a broad range of temperatures (Figure 5). One isolate exhibits robust growth at 30°C. Preliminary results suggest that this extragenic mutation lies within *POL30* (data not shown) and the basis for this interaction will be pursued under separate cover. To confirm our results that *elg1* fails to provide addition benefit to *ctf7 smc3^Q^* cells, we analyzed four additional isolates from an independent cross. The resulting *ctf7 elg1 smc3^Q^* triple mutant cells again exhibited growth equivalent to both *ctf7 elg1* and *ctf7 smc3^Q^* double mutant cells (not shown). These results support the model that Elg1-RFC directly opposes Ctf7 acetylation reactions, consistent with *in vitro* binding of Ctf7 to RFC complexes [23].

**Figure 6:**
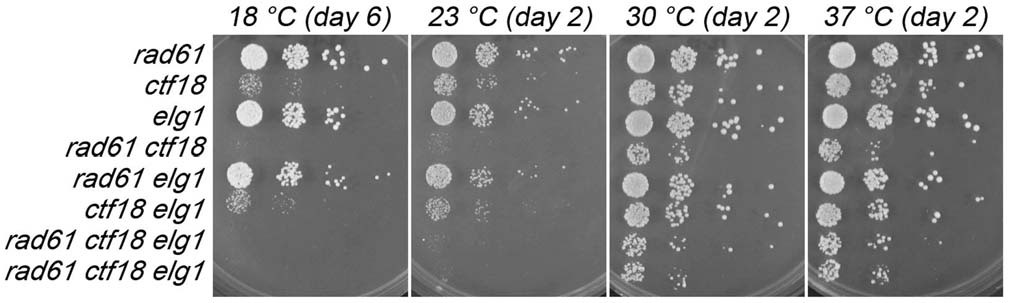
*rad61* deletion fails to provide growth benefit to either *elg1* or *ctf18* mutant cells. 10 fold serial dilutions of *rad61*, *ctf18* and *elg1* single mutant cells, *rad61 ctf18*, *rad61 elg1* and *ctf18 elg1* double mutant cells and also two independent isolates of *rad61 ctf18 elg1* triple mutant cells. Colony growth shown for cells on rich medium plates grown at 18°C, 23°C, 30°C, and 37°C for number of days indicated. Strains shown include YMM808, YBS1159, YMM207, YMM812, YMM818, YMM298, YMM816 and YMM817.

Pair-wise combinations provide a third route from which we could further address fundamental questions regarding the mechanisms through which anti-establishment factors (Rad61 and Elg1-RFC) function and through which a pro-establishment factor (Ctf18-RFC) functions. We crossed *rad61*, *ctf18* and *elg1* deletion cells and sporulated the resulting heterozygous diploid strains to obtain the range of double and triple mutant cells. We started from the observation that deletion of either *rad61* or *elg1* partially rebalances the cohesion defect of *ctf7* mutant cells mutants. If Rad61 and Elg1-RFC function through a common mechanism, then *rad61 elg1* double mutant cells should exhibit growth kinetics similar to either single mutant. If Rad61 and Elg1-RFC act through parallel mechanisms, then deletion of both genes should produce non-overlapping deficiencies observable as exacerbated growth defects. In fact, the growth of *rad61 elg1* double mutants is indistinguishable from that of either single mutant strain across a broad range of temperatures (18°C, 23°C, 30°C and 37°C) (Figure 6). These findings support a single mechanism of anti-establishment activity through which cohesin-associated and DNA replication fork-associated pathways may converge. We further note that *ctf18* deletion rendered each genetic combination (*ctf18 ctf7*, *ctf18 ctf7 rad61* and *ctf18 ctf7 rad61 elg1*) severely growth compromised or inviable (Figure 6), supporting a unique pro-establishment role for Ctf18-RFC.

**Figure 7:**
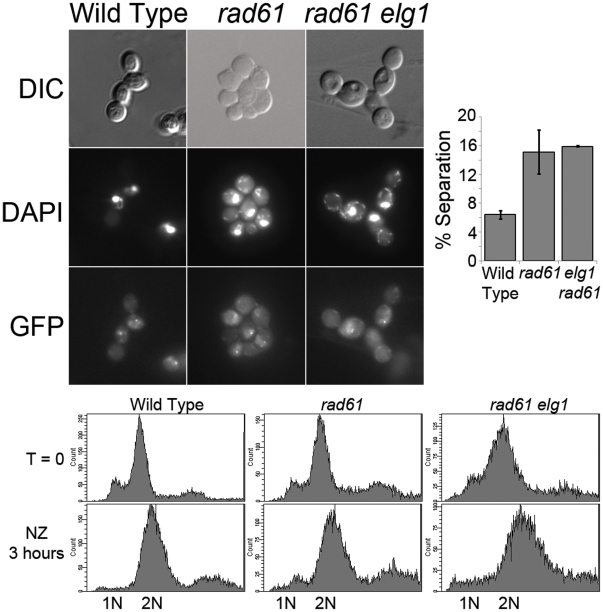
Cohesion assays comparing wildtype, *rad61* and *elg1* single mutant cells and *rad61 elg1* double mutant cells. Top left: Differential Interference Contrast (DIC) microscopy epi-fluorescence microscopy images highlight cell morphology and co-localization of DNA (DAPI) and sister chromatid loci (GFP). Bottom: DNA content profiles obtained by flow cytometry. Top right: Quantification of sister chromatid pairing defects (see Materials and Methods). Error bars represent max and min of each trial. Strains shown include YMM334, YMM985 and YMM988.

A fourth prediction of the hypothesis that Rad61 and Elg1-RFC regulate cohesion through a singular mechanism is that *rad61 elg1* double mutant cells should exhibit cohesion defects identical to either single mutant strain. To test this directly, wildtype, *rad61* single mutant cells and *rad61 elg1* double mutant cells were crossed into a cohesion assay strain in which *TetO* arrays are integrated approximately 40 kb from centromere V and detected via constitutive expression of GFP-tagged *TetR-GFP TetO*-binding protein. Log phase cultures were then split and arrested in pre-anaphase using nocodazole. We then counted the incidence of single (paired sisters) and two GFP spots (precociously separated sister chromatids) in pre-anaphase cells for each culture. As expected, wildtype cells contained predominantly paired sisters (∼6% separated sisters). In contrast, *rad61* mutant cells exhibit significant cohesin defects (∼15%), nearly identical to the cohesion defect detected in *elg1* mutant cells [27]. These pairing defects are not additive, however, in that *rad61 elg1* double mutant cells exhibit pairing defects indistinguishable from that of *rad61* single mutant cells (Figure 7).

### PCNA functions separately from that of Ctf7 acetylation of Smc3

**Figure 8:**
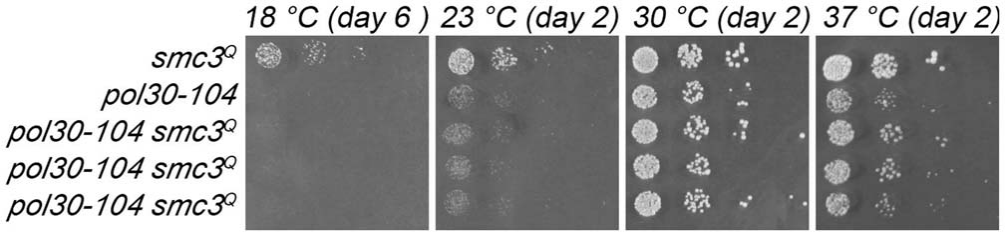
Expression of *smc3* acetylmimetic in *pol30-104* mutant cells. 10 fold serial dilutions of *smc3^Q^* and *pol30-104* single mutant cells and three independent isolates of *smc3^Q^ pol30-104* double mutant cells. Colony growth shown for cells on rich medium plates grown at 18°C, 23°C, 30°C, and 37°C for number of days indicated. Strains shown include YMM890, YMM891, YMM892, YMM893 and YMM894.

Cells that harbor mutations in PCNA (*pol30-104*) exhibit cohesion defects and are lethal in combination with *ctf7* [7], [21]. At face value, these observations suggest that PCNA might exert a role on Ctf7-dependent Smc3 acetylation. We decided to test two specific predictions of this model: that *smc3^Q^* would rescue *ctf7 pol30* lethality and that *smc3^Q^* would suppress *pol30* cell growth defects. *ctf7-203 smc3^Q^* double mutant cells were crossed to *pol30-104* cells and the resulting diploids sporulated. As expected, we recovered the appropriate number of single mutant *ctf7*, *pol30* and *smc3^Q^* single mutant spores and failed to recover any *ctf7 pol30* double mutant spores. In contrast to the first prediction, *ctf7 pol30 smc3^Q^* triple mutant spores were never recovered despite numerous independent crosses and sporulations (Table 3). These findings raise the possibility that *pol30 ctf7* lethality does not arise from loss of Smc3 acetylation. The second prediction that *smc3^Q^* would rescue *pol30* mutant cell cohesion-based growth defects also proved to be false. The results show that *pol30 smc3^Q^* double mutant cells exhibit growth kinetics nearly identical to that of *pol30* single mutant cells – no rescue was discernible at any temperature tested (Figure 8). Nor did *pol30 smc3^Q^* double mutant cells exhibit additional growth defects beyond those evident in *pol30* single mutant cells. In combination, these results suggest that PCNA functions in cohesion parallel to but separate from that of Ctf7 acetylation of Smc3 and that *smc3^Q^* does not adversely affect DNA replication processes in a significant fashion.

**Table 3:** *smc3* acetyl mimics can not bypass *ctf7-203 pol30-104* synthetic lethality.

	Observed	Expected
Wild Type	10	14
*ctf7-203*	14	14
*pol30-104*	11	14
*smc3^K113Q^*	13	14
*ctf7-203 smc3^K113Q^*	9	14
*pol30-104 smc3^K113Q^*	10	14
*ctf7-203 pol30-104*	0	14
*pol30-104 ctf7-203 smc3^K113Q^*	0	14
Dead	45	0

Cells harboring *ctf7-203* mutation along with *smc3^K113Q^* acetyl mimic were crossed to cells carrying the *pol30-104* allele. Diploids were sporulated, dissected and tetrads analyzed. Genotypes obtained from this cross are located in the observed column. Results reflect analysis from strain YMM697 crossed to strain CH2161.

**Figure 9:**
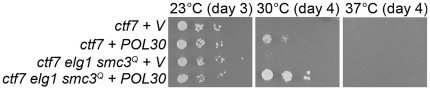
Effects of *POL30* (PCNA) over-expression in *ctf7-203* (*ctf7*) single mutant cells and *ctf7-203 elg1 smc3^Q^* triple mutant cells. Vector control plasmid (V) also shown. 10 fold serial dilutions of log phase growth cells plated onto selective medium shown after growth at 23°C and 30°C. Strains shown include YMM918, YMM919, YMM920 and YMM921.

To test further the model that PCNA (*POL30*) functions in cohesion separate from Ctf7, we capitalized on findings that *ctf7* mutant cell phenotypes are suppressed by *POL30* over-expression [9]. If PCNA indeed functions parallel but separate to Ctf7 and thus Smc3 acetylation, we hypothesized that *POL30* over-expression should provide added growth benefits to *ctf7* mutant cells beyond that of either *elg1* or *smc3^Q^*. To test this predication, *ctf7* single mutant and *ctf7 elg1 smc3^Q^* triple mutant cells were transformed either with vector alone or vector providing for elevated PNCA expression. As expected, *ctf7* mutant cells are growth inhibited at 30°C while *ctf7* mutant cells harboring elevated levels of PCNA exhibit modest growth at this temperature (Figure 9). The combination of *elg1* and *smc3^Q^* failed to provide modest growth to *ctf7* mutant cells at 30°C. In contrast, elevated PCNA levels combined with *elg1* and *smc3^Q^* provided for robust growth of *ctf7* mutant cells up to 30°C (Figure 9). This combination is unable to bypass *ctf7* mutant cell inviability at 37°C.

## Discussion

Only in the last year has a more complete accounting of sister chromatid cohesion anti-establishment factors become clear [16]. Two classes have become evident: cohesin-associated factors (Rad61/WAPL and Pds5) and DNA replication fork-associated complexes (Elg1-RFC). In the current study, we show that the anti-establishment activities of these two classes are genetically non-additive. One interpretation of these findings is that anti-establishment factors may work through the same pathways such that Elg1-RFC and Rad61 both inhibit Ctf7. Based on this, we favor a model that Rad61 and Elg1-RFC work in concert to oppose cohesion establishment and that these anti-establishment dynamics occur in concert as DNA fork components interact with cohesins. Moreover, our data supports a model that Elg1-RFC anti-establishment activity occurs via regulating Ctf7-dependent Smc3 acetylation. Likely scenarios are that Elg1-RFC 1) binds and sequesters Ctf7 to inhibit its acetyltransferase activity, 2) enhances the anti-establishment activities of other factors or 3) moves with the DNA replication fork to directly regulate cohesin complexes loaded during replication. All three are consistent with prior findings that Ctf7 physically associates with RFCs in vitro [20].

**Figure 10:**
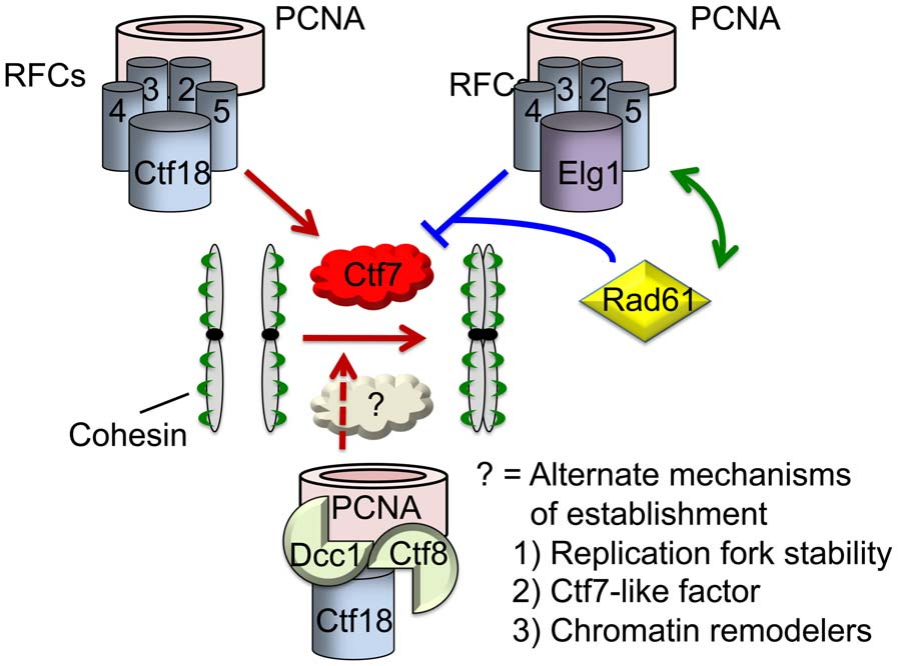
Model of anti-establishment (Elg1-RFC) and pro-establishment (Ctf18-RFC and Ctf18-Dcc1-Ctf8) complexes. Two pro-establishment pathways are described, one of which occurs independent of Ctf7-dependend acetylation of Smc3. Several speculative mechanisms are highlighted. In contrast, Elg1-RFC and Rad61 may function through a singular pathway. See text for details.

Pro-establishment replication factors include, but are not limited to, PCNA, Ctf18-RFC and Chl1 [21], [25], [26], [29]–[30], [32]. Beyond identification, little is known regarding pro-establishment mechanisms. This current study provides important insights regarding the roles of both Ctf18-RFC and PCNA in cohesion. As opposed to models in which PCNA recruits/activates Ctf7, a number of findings suggest instead that both Ctf18-RFC and PCNA promote cohesion establishment in addition to Ctf7-dependent Smc3 acetylation. Our finding that *ctf7 ctf18* double mutant cell lethality can be bypassed by additional deletion of *RAD61* indicates that *ctf7 ctf18* lethality is a result of cohesion defects and not severe DNA replication defects since *rad61* deletion specifically reduces *ctf7* mutant cohesion defects [36]. In combination with our data that Smc3 acetylmimetics fail to bypass *ctf7 ctf18* cell lethality, we propose a more plausible scenario in which Ctf8-RFC, in addition to exhibiting roles DNA replication, functions in cohesion separately from Ctf7 (Figure 10). For example, Ctf18 may aid in modifying DNA/chromatin or cohesion complexes for proper establishment to occur. This model is consistent with prior studies indicating that several chromatin remodeling complexes (RSC and INO80) play important roles in cohesion pathways [1], [41]–[43].

In combination, the findings reported here reveal an even more complex role for replication fork RFC proteins in cohesion establishment beyond PCNA dynamics and strongly suggest that current popular models must be thoroughly revised to reflect the truly complicated nature of these processes.

## Methods

### Yeast strains, plasmids and media

All strains used in this study were performed in the indicated backgrounds (Table S1). Media used for growth and sporulation are described previously [44]. *POL30* constructs are described previously [9]. To construct *rad61* knockout cells, PCR fragments were generated using AGAGAAACTATCGCAAAACGAAACCATCTTCTTACCCTAAAGCATCCTGTTTCTGAAAAAGATTGTACTGAGAGTGCACCATAC and TTTTCAATAGTTGCCAGCAGGGTGAAGATGAAGCCAGGCTATGTTCAATGTATGCTTTCTCTATTCTTTTGATTTATAAGGGAT with a *URA3* integrating vector and transformed into S288C strain that contains a mutated *URA3* gene. Proper integration was confirmed by using primers GAGTAGCATTACGTTTAGCCA and AAAGATCCTGGTAGCTTCAAT.

### Flow cytometry

Log phase cultures maintained at 30°C were normalized to an optical density between 0.1 and 0.25. Cells were arrested in YPD supplemented with alpha factor (5 µg/ml final concentration) for 3 hours at 30°C. Alpha factor was washed out and cells rinsed with pre-warmed YPD followed by incubation in YPD for 2 hours at 37°C. Samples were collected for Flow Cytometry analysis every 15 minutes by fixing cells in 0.2 M Tris 70% EtOH solution. Cells were then treated with RNase (Roche) and proteinase K (Roche) solutions to remove RNA and protein, respectively. To analyze DNA content, cells were stained with a 0.0001% propidium iodide (Sigma) solution (1000X stock generated by suspending 13 mg PI into 8.6 ml H_2_O. Prior to use, this stock is diluted 10 ul +990 ul of Tris solution for each milliliter of sample). Cells were sonicated and DNA content quantified by flow cytometry using a BD FACSCanto II.

### Cohesion assay

Log phase cultures of wildtype and mutant cells were normalized to optical densitiy between 0.15 and 0.2 and shifted to fresh medium containing 20 µg/ml nocodazole for 3 hours at 23°C. Samples were collected for Flow Cytometry analysis (See above) and for cell morphology and GFP detection following paraformaldehyde fixation (10% paraformaldehyde for 10 minutes at 23°C). Large budded cells containing condensed nuclei (visualized by DAPI staining) were analyzed. Cells were visualized using IPLab software and digital images captured from a Nikon Eclipse E800 microscope. Cohesion analyses were repeated two times and a total of at least 200 cells counted.

## Supporting Information

Table S1 **Strains used in this study.** All strains are S288C background except where noted (* denotes A364A; ^#^ denotes W303).
(0.09 MB DOC)
