# Supplementary material for: Replication Factor C Complexes Play Unique Pro- and Anti-Establishment Roles in Sister Chromatid Cohesion
Source: PLoS One. 2010 Oct 27;5(10):e15381. doi: 10.1371/journal.pone.0015381 (PMC2965161; doi:10.1371/journal.pone.0015381)
Supplement: Table S1 — Strains used in this study. All strains are S288C background except where noted (* denotes A364A; # denotes W303). (0.09 MB DOC) [file pone.0015381.s001.doc]

Table S1: Strains

| CH2161 | *MATa pol30-104:LEU2 ura3-52 leu2-3,112* | Ref 40 |
| --- | --- | --- |
| YMM506* | *MATa ctf7-203 pHIS3-GFP-LacI-HIS3::his3-11,15 leu2-3,112 ura-52::smc3^K113Q^ bar1GAL lys4::LacO:NAT* | Ref 12 |
| YMM697* | *MATalpha ctf7-203 smc3^K113Q^:URA3 HIS3-GFP-LacI-HIS3::his3-11,15 lys4::LacO:NAT leu2-3,112 trp1-1* | This study |
| YMM705* | *MATalpha ctf18::LEU2 HIS3-GFP-LacI-HIS3::his3-11,15 leu2-3,112 ura3-52 bar1 lys4::LacO:NAT* | This study |
| YMM737* | *MATalpha ctf7-203 elg1::KAN smc3^K113Q^:URA3 lys4::LacO:NAT leu2-3,112 trp1-1* | This study |
| YMM739* | *MATalpha ctf7-203 elg1::KAN smc3^K113Q^:URA3 HIS3-GFP-LacI-HIS3::his3-11,15 lys4::LacO:NAT leu2-3,112 trp1-1* | This study |
| YMM784* | *MATa ctf18::LEU2 smc3^K113Q^:URA3 HIS3-GFP-LacI-HIS3::his3-11,15 lys4::LacO:NAT leu2-3,112* | This study |
| YMM865 | *MATalpha ctf7-203 ade2-101 his3∆200 leu2 lys2-801 trp1∆63 ura3-52* | This study |
| YMM866 | *MATalpha ctf7-203 elg1::KAN ade2-101 his3∆200 leu2 lys2-801 trp1∆63 ura3-52* | This study |
| YMM867 | *MATalpha ctf7-203 smc3^K113Q^:URA3 ade2-101 his3∆200 leu2 lys2-801 trp1∆63 ura3-52* | This study |
| YMM868 | *Bimater ctf7-203 elg1::KAN smc3^K113Q^:URA3 ade2-101 his3∆200 leu2 lys2-801 trp1∆63 ura3-52* | This study |
| YMM869 | *MATa ctf7-203 elg1::KAN smc3^K113Q^:URA3 ade2-101 his3∆200 leu2 lys2-801 trp1∆63 ura3-52* | This study |
| YMM870 | *MATalpha ctf7-203 elg1::KAN smc3^K113Q^:URA3 ade2-101 his3∆200 leu2 lys2-801 trp1∆63 ura3-52* | This study |
| YMM871 | *MATalpha ctf7-203 elg1::KAN smc3^K113Q^:URA3 ade2-101 his3∆200 leu2 lys2-801 trp1∆63 ura3-52* | This study |
| YMM872 | *MATalpha smc3^K113Q^:URA3 ade2-101 his3∆200 leu2 lys2-801 trp1∆63 ura3-52* | This study |
| YMM873 | *MATalpha ctf18::LEU2 ade2-101 his3∆200 leu2 lys2-801 trp1∆63 ura3-52* | This study |
| YMM874 | *MATa ctf18::LEU2 smc3^K113Q^:URA3 ade2-101 his3∆200 leu2 lys2-801 trp1∆63 ura3-52* | This study |
| YMM875 | *MATalpha ctf18::LEU2 smc3^K113Q^:URA3 ade2-101 his3∆200 leu2 lys2-801 trp1∆63 ura3-52* | This study |
| YMM876 | *MATa ctf18::LEU2 smc3^K113Q^:URA3 ade2-101 his3∆200 leu2 lys2-801 trp1∆63 ura3-52* | This study |
| YMM890 | *MATa smc3^K113Q^:URA3 ade2-101 his3∆200 leu2 lys2-801 trp1∆63 ura3-52* | This study |
| YMM891 | *MATalpha pol30-104:LEU2 ade2-101 his3∆200 leu2 trp1∆63 ura3-52* | This study |
| YMM892 | *MATa pol30-104:LEU2 smc3^K113Q^:URA3 ade2-101 his3∆200 leu2 trp1∆63 ura3-52* | This study |
| YMM893 | *MATalpha pol30-104:LEU2 smc3^K113Q^:URA3 ade2-101 his3Δ200 leu2 lys2-801 trp1∆63 ura3-52* | This study |
| YMM894 | *MATa pol30-104:LEU2 smc3^K113Q^:URA3 ade2-101 his3∆200 leu2 trp1∆63 ura3-52* | This study |
| YMM808 | *MATa rad61::URA3 ade2-101 his3∆200 leu2∆1 lys2-801 trp1∆63 ura3-52* | This study |
| YJE105 | *MATalpha ctf18::LEU2 ade2-101 his3∆200 leu2∆1 lys2-801 trp1∆63 ura3-52* | Ref 45 |
| YJE106 | *MATa ctf18::LEU2 ade2-101 his3Δ200 leu2∆1 lys2-801 trp1∆63 ura3-52* | Ref 45 |
| YMM207 | *MATalpha elg1::KAN ade2-101 his3∆200 leu2∆1 lys2-801 trp1∆63 ura3-52* | This study |
| YMM812 | *MATalpha rad61::URA3 ctf18::LEU2 ade2-101 his3∆200 leu2∆1 lys2-801 trp1∆63 ura3-52* | This study |
| YMM818 | *MATalpha rad61::URA3 elg1::KAN ade2-101 his3∆200 leu2∆1 lys2-801 trp1∆63 ura3-52* | This study |
| YMM298 | *MATa ctf18::LEU2 elg1::KAN ade2-101 his3∆200 leu2∆1 lys2-801 trp1∆63 ura3-52* | This study |
| YMM816 | *MATalpha rad61::URA3 ctf18::LEU2 elg1::KAN ade2-101 his3∆200 leu2∆1 lys2-801 trp1∆63 ura3-52* | This study |
| YMM817 | *MATalpha rad61::URA3 ctf18::LEU2 elg1::KAN ade2-101 his3∆200 leu2∆1 lys2-801 trp1∆63 ura3-52* | This study |
| YMM828 | *MATalpha cft7::HIS3 rad61::URA3 ade2-101 his3∆200 leu2∆1 lys2-801 trp1∆63 ura3-52* | This study |
| YMM829 | *MATa cft7::HIS3 rad61::URA3 ade2-101 his3∆200 leu2∆1 lys2-801 trp1∆63 ura3-52* | This study |
| YMM821 | *MATalpha cft7::HIS3 rad61::URA3 elg1::KAN ade2-101 his3∆200 leu2Δ1 lys2-801 trp1Δ63 ura3-52* | This study |
| YMM823 | *MATa cft7::HIS3 rad61::URA3 elg1::KAN ade2-101 his3Δ200 leu2Δ1 lys2-801 trp1Δ63 ura3-52* | This study |
| YMM820 | *MATa cft7::HIS3 rad61::URA3 ctf18::LEU2 ade2-101 his3Δ200 leu2Δ1 lys2-801 trp1Δ63 ura3-52* | This study |
| YMM825 | *MATa cft7::HIS3 rad61::URA3 ctf18::LEU2 ade2-101 his3Δ200 leu2∆1 lys2-801 trp1∆63 ura3-52* | This study |
| YMM827 | *MATalpha cft7::HIS3 rad61::URA3 ctf18::LEU2 ade2-101 his3∆200 leu2∆1 lys2-801 trp1∆63 ura3-53* | This study |
| YMM822 | *MATalpha cft7::HIS3 rad61::URA3 ctf18::LEU2 elg1::KAN ade2-101 his3∆200 leu2∆1 lys2-801 trp1∆63 ura3-53* | This study |
| YMM824 | *MATa cft7::HIS3 rad61::URA3 ctf18::LEU2 elg1::KAN ade2-101 his3∆200 leu2∆1 lys2-801 trp1∆63 ura3-53* | This study |
| YBS255 | *MATa CTF7:LEU2 ctf7::HIS3 ade2-101 his3∆200 leu2∆1 lys2-801 trp1∆63 ura3-53* | Ref 9 |
| YBS514 | *MATa ctf7-203 ctf7::HIS3 ade2-101 his3∆200 leu2∆1 lys2-801 trp1∆63 ura3-53* | Ref 9 |
| YMM813 | *MATa rad61::URA3 ctf18::LEU2 ade2-101 his3∆200 leu2∆1 lys2-801 trp1∆63 ura3-52* | This study |
| YMM334^#^ | MATa ade2-1 his3-11,15 leu2-3,112 trp1-1 ura3-1 CTF7:ADE2 URA3:tetO LEU2:tetR-GFP TRP1:PDS1-MYC13 | Ref 27 |
| YMM985^#^ | MAT*alpha* ade2-1 his3-11,15 leu2-3,112 trp1-1 ura3-1 URA3:tetO LEU2:tetR-GFP TRP1:PDS1-MYC13 rad61::URA3 | This study |
| YMM988^#^ | MATa ade2-1 his3-11,15 leu2-3,112 trp1-1 ura3-1 URA3:tetO LEU2:tetR-GFP TRP1:PDS1-MYC13 rad61::URA elg1::KAN | This study |
| YMM918 | *MATalpha ctf7-203 ade2-101 his3∆200 leu2 lys2-801 trp1∆63 ura3-52 (pRS315 = CEN LEU2 vector)* | This study |
| YMM919 | *MATalpha ctf7-203 ade2-101 his3∆200 leu2 lys2-801 trp1∆63 ura3-52 (pBS104 = CEN LEU2 POL30)* | This study |
| YMM920 | *MATalpha ctf7-203 elg1::KAN smc3^K113Q^:URA ade2-101 his3∆200 leu2 lys2-801 trp1∆63 ura3-52 (pRS315 = CEN LEU2 vector)* | This study |
| YMM921 | *MATalpha ctf7-203 elg1::KAN smc3^K113Q^:URA ade2-101 his3∆200 leu2 lys2-801 trp1∆63 ura3-52 (pBS104 = CEN LEU2 POL30)* | This study |
